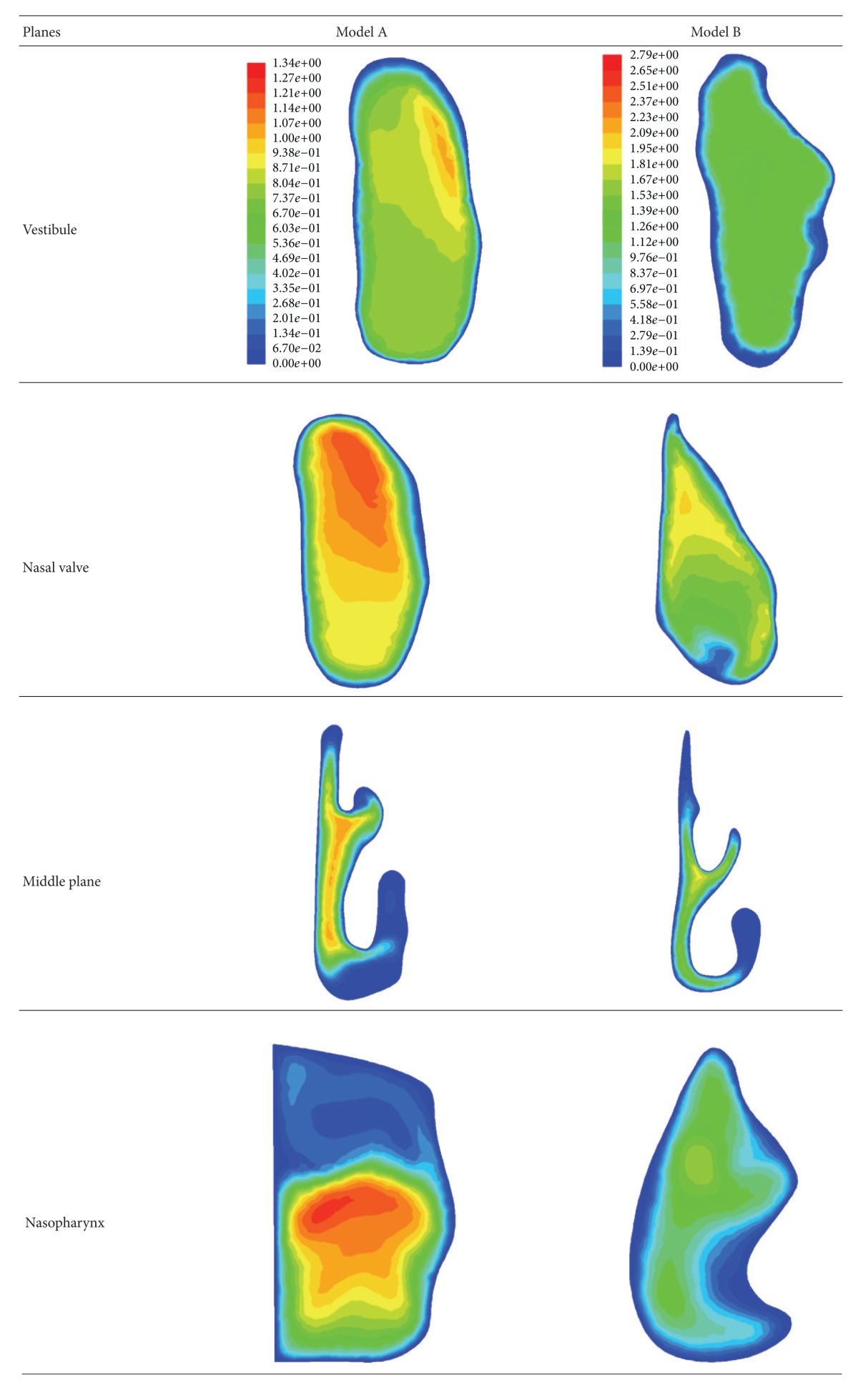# Standardization of Malaysian Adult Female Nasal Cavity

**DOI:** 10.1155/2013/519071

**Published:** 2013-06-15

**Authors:** Chih Fang Lee, Mohd. Zulkifly Abdullah, Kamarul Arifin Ahmad, Ibrahim Lutfi Shuaib

**Affiliations:** ^1^School of Aerospace Engineering, Engineering Campus, Universiti Sains Malaysia, 14300 Nibong Tebal, Pulau Pinang, Malaysia; ^2^School of Mechanical Engineering, Engineering Campus, Universiti Sains Malaysia, 14300 Nibong Tebal, Pulau Pinang, Malaysia; ^3^Department of Aerospace Engineering, Universiti Putra Malaysia, 43400 Serdang, Selangor, Malaysia; ^4^Advanced Medical & Dental Institute, Universiti Sains Malaysia, 13200 Kepala Batas, Pulau Pinang, Malaysia

## Abstract

This research focuses on creating a standardized nasal cavity model of adult Malaysian females. The methodology implemented in this research is a new approach compared to other methods used by previous researchers. This study involves 26 females who represent the test subjects for this preliminary study. Computational fluid dynamic (CFD) analysis was carried out to better understand the characteristics of the standardized model and to compare it to the available standardized Caucasian model. This comparison includes cross-sectional areas for both half-models as well as velocity contours along the nasal cavities. The Malaysian female standardized model is larger in cross-sectional area compared to the standardized Caucasian model thus leading to lower average velocity magnitudes. The standardized model was further evaluated with four more Malaysian female test subjects based on its cross-sectional areas and average velocity magnitudes along the nasal cavities. This evaluation shows that the generated model represents an averaged and standardized model of adult Malaysian females.

## 1. Introduction

The human nasal cavity consists of two symmetrically complex three-dimensional nasal passages that are separated in the middle by the nasal septum. During inspiration, air flows into the nasal cavity from the nostrils and then reaches the smallest cross-sectional area, the nasal valve, before reaching the tortuous turbinates region that forms large cross-sectional areas covered with mucous layers and cilia. These moist regions play an important role for the humidification, warming, and cleaning of the inspired air by entrapping the air-borne particles as well as moistening the air by evaporation [1]. Then, the turbinates region will guide the airflow towards the posterior region of the nasal cavity which is the nasopharynx. 

Objective measurement methods are also very common in studies related to nasal geometry where it is used to determine cross-sectional area nasal airway resistance, and also visualization of the human nose. Shelton and Eiser carried out an evaluation of active anterior and posterior rhinomanometry in normal subjects [2]. Suzina et al. used active anterior rhinomanometry (AAR) for the objective assessment of the nasal airway resistance in normal adult Malays [3]. However, these methods have their limitations in measuring the precise velocity of the airflow as well as in evaluating the local nasal resistance in every portion of the nasal cavities [4]. In addition to that, the complex nasal anatomy consists of numerous thin airway channels that prevent direct experimental measurements of the flow patterns inside the nose [5]. 

In recent years, with the rapid development in computer resources, there have been increasingly wide and deep applications of the CFD technique to studying the airflow characteristics in the human nasal cavity and hence its correlation with the symptoms and functions of the human nose [6–11]. Majority of these studies used a combination of different softwares (e.g., Mimics, Amira, and ICEM-CFD) to produce numerical nasal cavity models. In addition to that, there are a few review papers in this area, such as Bailie et al. [12], Leong et al. [13], and Zubair [14]. A better understanding of the nose physiology, pathophysiology during normal breathing, and the postprocessing techniques of the flow patterns in nasal cavities can be achieved using CFD [15–17]. CFD was implemented to study the various aspects of respiratory airflow generated from the branching network tubes that make up the tracheal-bronchial tree. The model developed from the study of Lai et al. was able to provide an overall insight into the effect of fluid flow in the human upper respiratory airways [18]. Wide range of CFD applications were not only limited to upper respiratory airflow as Do et al. utilized CFD to analyse the three-dimensional haemodynamics of a typical stenotic coronary artery bypass grafting (CABG). The study demonstrated how numerical investigation can give an insight into the haemodynamic of various configurations of CABG under various physiological conditions [19].

**Figure 1:**
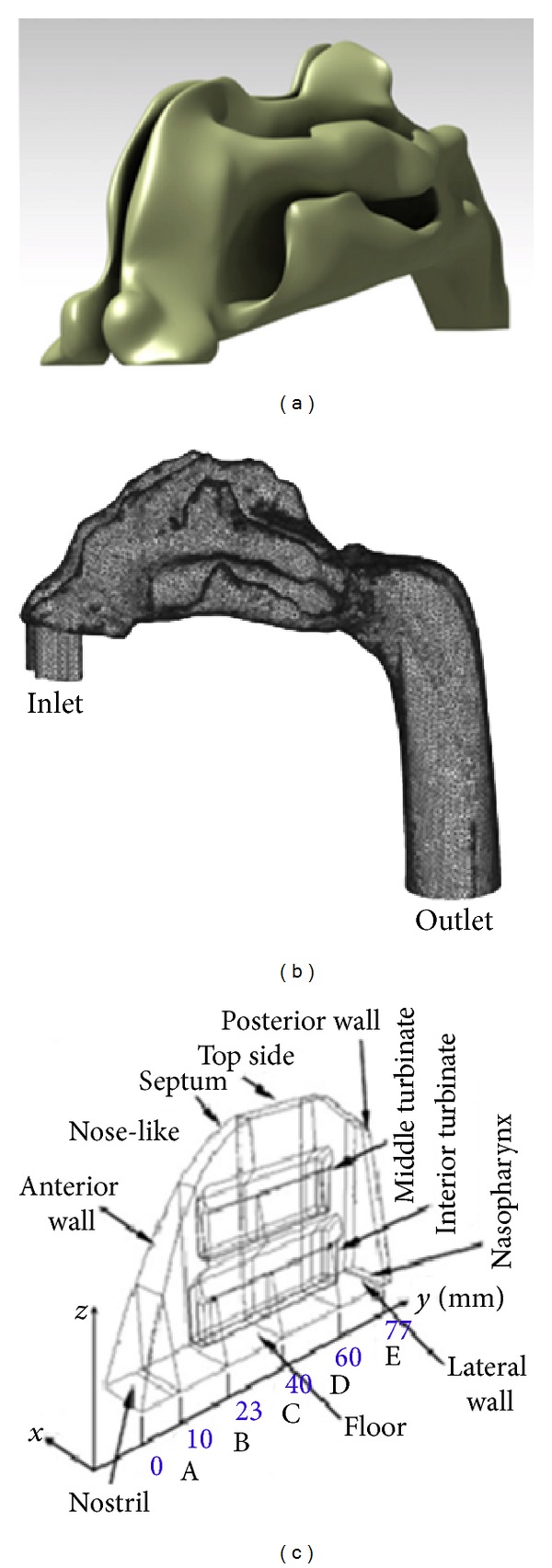
Models of the nasal cavity: (a) Anatomically identical model, (b) nasal cavity with additional inlet tube and extended nasopharynx, and (c) simplified nose-like model (figures obtained from Shi et al. [20] and Elad et al. [6]).

Generally, researchers used identical models as shown in Figure 1(a) as they replicate the exact structures of the nasal passageway, with only slight modifications to the structures for simplification purposes. Geometrical models varied with the type of studies performed. Therefore, models from a healthy human who does not possess any obvious pathological symptoms and vice versa are required for a better understanding and visualization of a normal nasal airflow [4, 10, 11, 16, 21, 22]. For example, Garcia et al. conducted a research using nasal cavity of a patient with primary atrophic rhinitis [1, 23]. This allows different conditions especially patients with chronic diseases to be analyzed and information to be obtained, which is vital in providing treatment for such cases. Furthermore, Croce et al. used a realistic plastinated human model which is anatomically conserved with left and right nasal cavities for experimental purposes. This model was scanned to obtain CT images for specific three-dimensional reconstruction procedures that were implemented for numerical simulations [24]. Results from both models were compared for validation purposes to ensure the accuracy of the analysis. A study on nanoparticle or vapour deposition which was carried out by Shi et al. required not only the typical anatomical model but also some additional geometry as shown in Figure 1(b). A short inlet tube was added to the nostril to prevent simple plug flow from entering the nostrils, and a certain length of actual airway was added to the nasopharynx to obtain proper outlet conditions [20]. 

Other than anatomically identical models, Elad et al. implemented a nose-like model as shown in Figure 1(c), which was a simplified model of the nose generated using average data of human nasal cavities [6]. The superior turbinate was omitted because the airflow in this region is very small. This generalized nose-like model simplified the complex structures of the three-dimensional nasal cavities and allowed removal or addition of various features to the model. These changes were very useful for the different types of comprehensive analysis to be carried out with ease [25]. For the analysis of air-conditioning capacity, the nose-like model was able to yield similar results to the anatomical model [26]. Hörschler et al. investigated the impact of the geometry on the nasal airflow by using different models of human nasal cavity with and without turbinates [27]. Although all these models were able to produce reliable data, these researches only focused on certain unique individuals, and the results do not represent general population. 

The interindividual difference of the unique characteristic of nasal cavity becomes a crucial issue in the comparison of results among different researches [17, 24, 28, 29]. Doorly et al. suggested that the establishment of rational methods to characterize and compare different anatomies would be very helpful in the future [8]. There is no guideline or benchmark that can be made as a reference when comparing the various results obtained. Therefore, this leads to the creation of a standardized model that will be used to represent a certain population. Liu et al. developed a method to scale, orient, and align the nasal geometries of 30 sets of CT scans of healthy subjects. The research also mentioned the importance of a standardized model for future experimental and numerical studies of inhaled aerosols [30]. 

The objective of this research is to create a standardized adult Malaysian female nasal cavity using a new approach that is simpler and applicable to a larger population. This standardized model was compared to existing standardized models from past researches to review the differences due to the different types of population based on geographical differences. Large differences especially in cross-sectional areas were observed from the Malaysian standardized model when compared to the Caucasian model. Thus, this proves that the standardized model is a good addition to the existing nasal models used in past researches.

## 2. Materials and Methods 

This paper discusses the new methods in generating the standardized model in details. This method is a new approach as it is different compared to the method implemented by Liu et al. [30]. A complete set of average images used to generate the standardized model can be created in less than a few hours, saving time and cost as well as human labour. Developing the CT scans into three-dimensional model requires extra detail especially in determining the boundary layer of the nasal cavity and strict guidance from a rhinologist. Generation of the standardized nasal geometry involves several important steps that can be divided into three major parts. The first part is construction of geometry, followed by meshing of geometry, and finally running and analyzing the results. For this research, the standardized Malaysian female nasal cavity was created, and analysis was carried out to understand and verify the averaged model.

**Figure 2:**
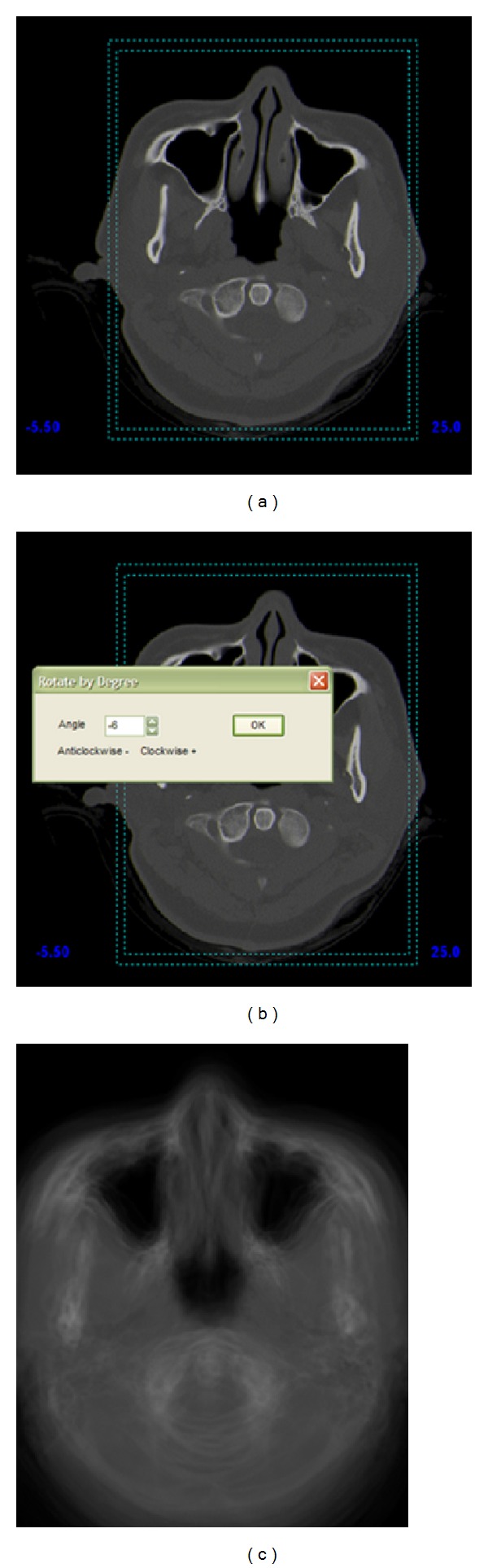
(a) Cropping image, (b) rotating image, and (c) average image obtained.

The first part of methodology involved generation of the standardized nasal cavity from two-dimensional CT scans of 26 sets of normal healthy Malaysian females' cavities that were obtained from the Advanced Medical Department of Universiti Sains Malaysia. The CT scans were transferred into MIMICS (Materialise, USA) to generate a three-dimensional model of the nasal cavity. Axial, coronal, and sagittal views of the nasal cavity were obtained from MIMICS, but only sets of axial images (captured from the anterior to the posterior of nasal cavity) were used in generating the averaged standardized nasal cavity. Axial images are able to show the complex geometry such as turbinates more clearly compared to other orientations. In order to generate the standardized model, an image processing program was executed to calculate the average pixel values in every axial image. The first axial images of all the 26 sets of CT scans were grouped together, and average values were calculated to generate the first axial image for the standardized model. These methods were repeated for all the 37 images per test subject. This is a slightly tedious process, but only a few minutes are required to complete an average axial image. This study used CT images of both left and right nasal cavities to produce the averaged images. Several important precautions need to be taken such as cropping of the images. Since the size of the human head varies from individual to individual, the dimension, resolution, and position of the nasal cavity were maintained. The dimensions were measured from the septum with a crop ratio of 4 : 3 using the automatic cropping function of image cropper as shown in Figure 2(a). Another important detail that requires attention is the orientation of the nasal cavity during the CT scan as the subjects tend to position their heads in different directions. The entire image was rotated to a certain angle as shown in Figure 2(b) which ensures that the straight nasal septum can be viewed to obtain accurate average pixel values. The nasal septum plays an important role as it acts as a reference line for cropping as well as rotation of the images. Therefore, subjects with septum deviations are omitted from this research with the help of a rhinologist. Only healthy female subjects were used in this study.  Figure 2(c) shows one of the final averaged images located in the middle of the nasal cavity from axial view. The different lines were created from all 26 images of different subjects. 

**Figure 3:**
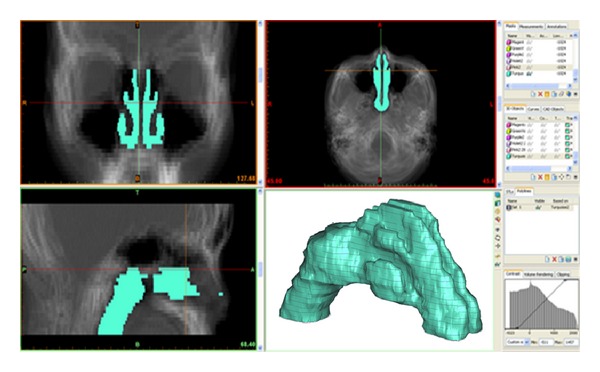
Creation of three-dimensional model in MIMICS.

A total of 37 new axial averaged images obtained from averaging of 26 test subjects were then imported to MIMICS to create a three-dimensional model as shown in Figure 3. Some functions like thresholding and editing the masks in MIMICS were used to facilitate the generation of the model. These functions were used to eliminate unwanted areas and to distinguish soft tissues and bone structures as well as empty spaces. The threshold in MIMICS neglected the soft tissues along nasal cavity as the nasal cavity was assumed to be decongested. The first draft of the standardized model can be seen in Figure 3 with some minor rough surfaces, but overall the structure seems to be well constructed. 

**Figure 4:**
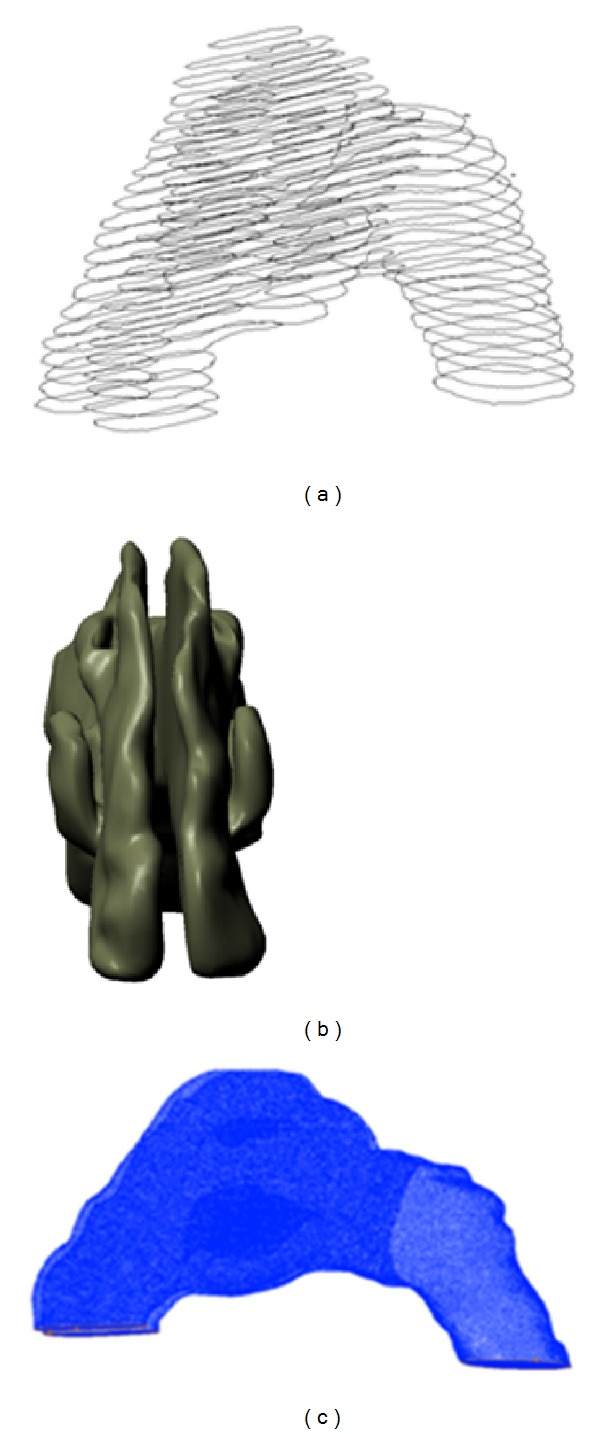
(a) Polylines from MIMICS, (b) smooth 3D nasal cavity from CATIA, and (c) meshing of geometry.

The three dimensional polylines data from MIMICS were then imported to CATIA V5 (Dassault Systémes) for smoothening the rough surfaces and to delete unwanted vertices.  Figure 4 shows how the standardized model is transformed from the raw polylines into the final standardized model that was used for analysis. All of the figures shown are actually just hollow surfaces. However, this model was converted into a fully solid volume for meshing purposes in GAMBIT 2.3.16 (Fluent Inc., Lebanon, USA).

**Figure 5:**
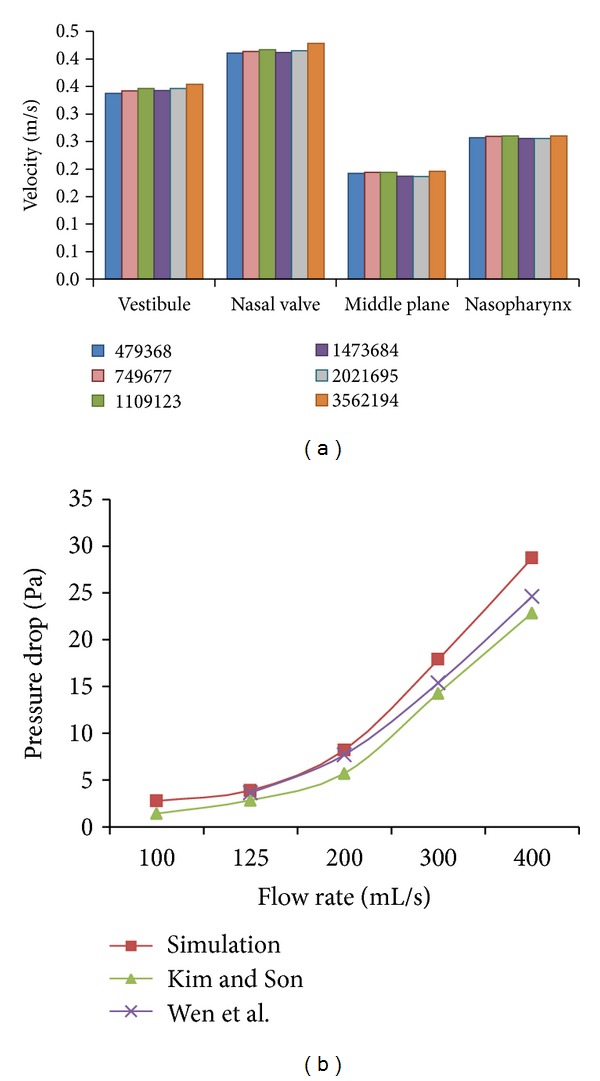
(a) Mesh dependency study at mass flow rate of 125 mL/s and (b) pressure drop versus mass flow rate.

Secondly, the methodology is focused on the meshing of the geometry. The standardized model generated from CATIA V5 was then imported to Gambit as a volume to perform volume meshing as shown in Figure 4(c). The grid independence study was carried out to determine the best mesh using unstructured tetrahedral meshing ranging from 500,000 to 3,000,000 elements. Lack of meshing elements especially at the thin turbinates region inside the nasal cavity will cause failure in capturing nasal airflow in the crucial areas. The results showed that the grid dependency study resulted in an optimized meshing of 1,109,123 elements, and the computational results are validated with the pressure drop obtained from Kim and Son [31] and Wen et al. [15, 17] as shown in Figure 5.

Finally, the last part of this methodology involved running the analysis and analyzing the results. The numerical simulation was performed using finite volume method provided by FLUENT 6.3.26 (Fluent, Lebanon, USA) for better understanding of the new standardized model. The simulation was based on the Navier-Stokes equations by representing the general equations for three-dimensional flow of incompressible and viscous fluids [28]. This study focused only on laminar airflow simulation for normal, resting breathing with a flow rate of 7.5 L min^−1^ [32, 33]. The boundary conditions defined were based on previous works [28, 34]. The nasal wall was assumed to be rigid, with no slip boundary condition, and effects of mucous were assumed to be negligible. The nostril inlet was defined by mass flow inlet, and the outlet at nasopharynx was defined by the outflow boundary condition. Any backflow at the outlet was assumed to be at 32.6°C and 100% relative humidity as imposed in FLUENT [1]. Nasal hair was also not considered as it is proven that it has no significant effect on the flow within the nasal cavity [35]. Paranasal sinuses were excluded in the creation of the standardized model as most researches (both computational and experimental as well as disease and nondisease cases) only focus on the study of nasal airflow and do not consider paranasal sinuses in analyses [1, 4, 6–8, 11, 15, 17, 21, 22, 24, 29, 30, 33, 34, 36–40]. The sinuses were deemed to have negligible impact on the gross airflow patterns due to the small openings (ostia) with minimal cross-sectional area [11, 24]. 

The same methodology was repeated for the generation and analysis of two half-models and four more female nasal cavities. One of the half-models was cut from the standardized model while the other was obtained from Liu et al. (generated from 30 sets of Caucasian nasal cavities). Both models were compared in order to view the differences between the standardized model of different populations based on geographical differences as well as the methods applied in their generation. Meanwhile, the four Malaysian female subjects were carefully chosen from the 26 test subjects based on their ages and races. These four models were used for comparison with the standardized model to prove that the generated standardized model represents an average model of a Malaysian female nasal cavity. The information for all the 26 subjects is presented in Table 1. It was previously mentioned that the methodology for the current study is suitable for a large population. However, only 26 subjects were asked to participate in this study as it is only a preliminary study. Another reason which limits the number of subjects for this study is the lack of data as most patients with nasal pain or diseases are only willing to do the CT scans. Therefore, it is harder to obtain samples of healthy cases for the current study. 

## 3. Results and Discussion

Results obtained from the standardized model generated from 26 female subjects were presented and discussed in two major parts. The first part is the comparison of two half-models; one is from the generated standardized model (labelled as Model A), and the other half-model is obtained from the research of Liu et al. [30] (labelled as Model B) for discussions of various results obtained from the CFD analysis. Comparisons were carried out by studying the cross-sectional areas, average velocity magnitudes, and contours coloured by velocities at four main cross-sections along the nasal cavities for both models. Based on the comparisons, variations among the standardized model of both geographically different nasal cavities can be obtained thus proving the requirement for a standardized model of different populations. The second part was only focused on the generated standardized model and the four nasal cavities chosen from the group of test subjects. Evaluation was carried out on all five complete models by comparing its cross-sectional areas and average velocity magnitudes at four cross-sections along the nasal cavities. The results obtained prove that the generated standardized model is able to represent the averaged adult Malaysian female nasal cavity.

### 3.1. Comparison of the Half-Models

**Figure 6:**
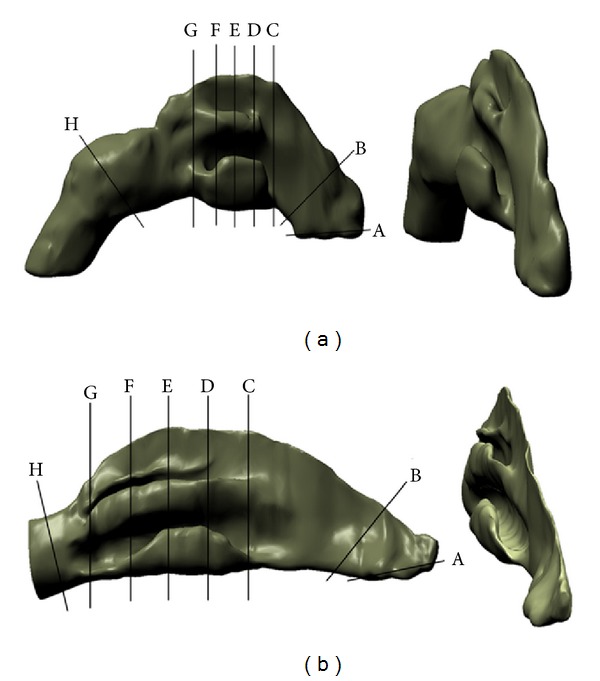
Half-models: (a) model from current study, Model A, and (b) model obtained from Liu et al. [30], Model B. Cutting planes: A = vestibule, B = nasal valve, C = plane 1, D = plane 2, E = middle plane, F = plane 3, G = plane 4, and H = nasopharynx.

The generated standardized model, Model A, is 99.312 mm in length which is relatively close to the average value presented in Table 1 while the half-model obtained from Liu et al., Model B, is 109.73 mm in length. Both models are shown in Figure 6 with obvious differences in the structures, both horizontally and vertically. Model B shows obvious existence of superior meatus, longer middle region of all turbinates, shorter nasopharynx, and an imprecise vestibule shape. On the other hand, Model A shows only inferior and middle meatuses, longer nasopharynx region, and a more accurate representation of the nasal vestibule. Longer nasopharynx region is more relevant to ensure proper outlet condition during CFD analysis [20]. Based on the observation made on all the 26 subjects, the inconsistent visibility of the superior meatuses caused Model A to consist only of superior and middle meatuses. Cutting planes as shown in Figure 6 were implemented to obtain the required information because of the variation of lengths among the models. These cutting planes allowed comparison to be carried out at certain locations along the nasal cavities. There are a total of 8 cutting planes with the first one being A, located at the vestibule which is slightly upward from the inlet. The second cutting plane B is located at the nasal valve, which is the smallest cross-section of the nasal cavity. The third cutting plane, plane 1, C is located at the starting of the inferior meatus while the middle plane E is located at the middle of all the meatuses and plane 4 G is located at the end of the inferior meatus. Both planes 2, D, and 3, F, are located in between of C–E and E–G, respectively. Finally, the nasopharynx H is located at the end of the nasal cavity near the outlet. 

**Figure 7:**
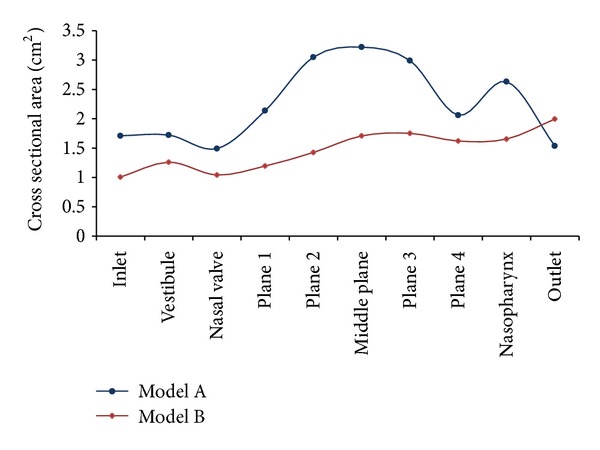
Cross-sectional areas along nasal cavities.

Cross-sectional areas along the nasal cavities are presented in Figure 7 for a more thorough comparison of both models. It is noticeable that Model B has a smaller cross-sectional area compared to Model A as shown in Figures 6 and 7. This is due to the more slender shape of Model B. The cross-sectional area of Model A is higher except for the outlet due to the longer nasopharynx region of the model. Model A shows the lowest cross-sectional area located at the nasal valve and the highest cross-sectional area located at the middle plane of the meatuses. The sudden decline on plane 4 was caused by the ending of the meatuses. Both models were obtained from different populations at different geographical locations thus causing the variation in the cross-sectional areas. From a visual observation, it seems that the Malaysian nose is comparatively smaller in size and length when compared to the Caucasian nose. However, the result indicated in Figure 7 clearly shows otherwise. This shows that the outer nose appearance cannot be used to estimate the cross-sectional areas of the inner nasal cavity. 

**Figure 8:**
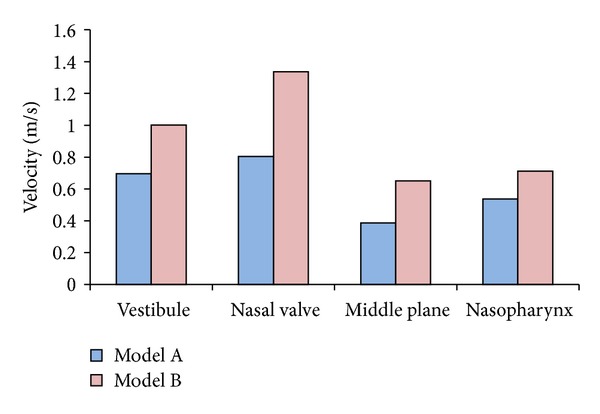
Graph of average velocity magnitudes at four cross-sections.

The capabilities of CFD to present useful information on nasal cavities are undeniable, as it has been presented by various researches for over a decade [21, 41–43]. For this paper, CFD analysis was carried out to further investigate the differences between both standardized models.  Figure 8 shows the graphs of average velocity magnitudes while the contours of velocities are illustrated in Table 2. Contours of velocities were chosen for discussion as they clearly show the physical differences of both models as well as the airflow analysis in the nasal cavities. Obvious differences were observed from both models as indicated in Figure 8 and Table 2. All this information was obtained from four main cross-sections along the nasal cavity, which are the vestibule, nasal valve, middle plane, and nasopharynx. Model A shows a vestibule and nasal valve that is more oval in shape while Model B shows an inconsistent shape. Thinner middle plane was observed for Model B compared to Model A, which showed a rounder shape of meatuses. Higher average velocity magnitudes were obtained for Model B for all cross-sections due to the smaller cross-sectional areas as indicated in Figure 6. On the other hand, lower average velocity magnitudes were obtained for Model A due to the generalized averaged model having a larger airway channel compared to Model B. Model B was created based on both female and male models while Model A only focused on Malaysian females. Similar patterns can be examined from both models as the highest velocity resulted from the nasal valve, which is the airflow restrictor before entering the meatus region. Increment in average velocity was observed from vestibule to nasal valve, which decreased at the middle plane and finally increased again at the nasopharynx as its cross-sections become smaller. Lower velocities were obtained from the middle plane regions as the meatuses function to enlarge the surface area exposed to the air. This increases the heat and moisture exchange inside the nasal cavity. The percentages of differences of velocity magnitudes between both models were relatively high, which are 30% for vestibule, 40% for nasal valve and middle plane, and 25% for nasopharynx. These big differences strongly support the importance of having a standardized model that represents different populations and to generate a standardized model based on a larger group of test subjects. 

### 3.2. Comparisons of Model A with 4 Other Female Models

**Figure 9:**
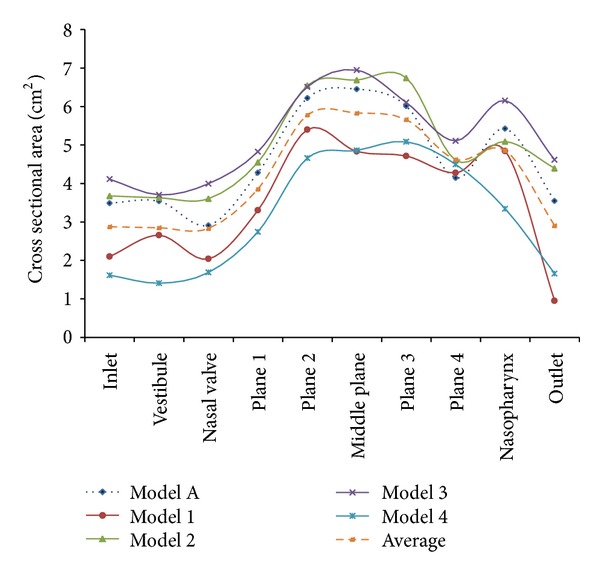
Cross-sectional area comparisons of Model A and other 4 models.

Comparisons made with Model B from the research of Liu et al. [30] are not sufficient to prove that Model A can be used to represent an averaged adult Malaysian female nasal cavity. Thus, further investigations were carried out by analysing four female models chosen from the group of 26 subjects by taking into consideration their races and age range. Only four models were chosen for the comparison.  Figure 9 shows the cross-sectional areas of all the five models and the average value calculated from the models. Model 1 and Model 4 seem to be smaller in size compared to Model A while Model 2 and Model 3 are slightly larger. The difference between Model A and the calculated average values is less than 20% for all cross-sections. Hence, this methodology was able to create a standardized model that is a very close approximation to the ideal average model. Based on the graphs, all the models possess similar patterns of cross-sectional areas. It was also noticed that an adult Malaysian female has relatively large vestibule and meatuses but a smaller nasopharynx. 

**Figure 10:**
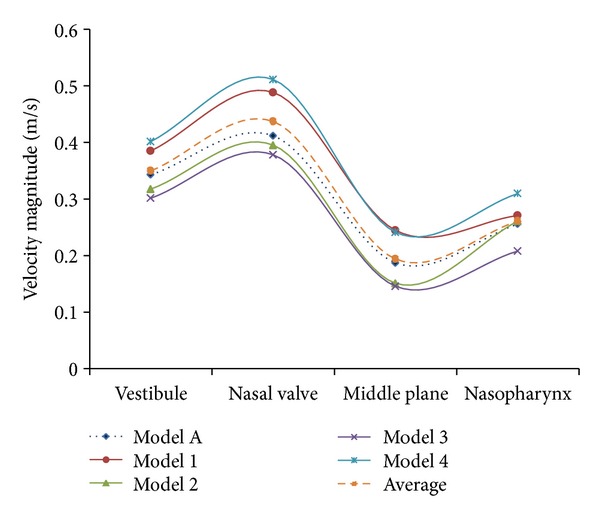
Graphs of average velocity magnitudes at four cross-sections along the nasal cavity.

Additional analysis was performed to enhance the understanding of this standardized Model A. It is noticed from Figure 10 that Model 1 and Model 4 possess higher average velocity magnitudes while Model 2 and Model 3 show lower average velocity magnitudes compared to Model A. This is due to the cross-sectional areas as indicated in Figure 9. Average velocity magnitudes obtained from Model A were very close to the average values from all the models. Similar patterns of all the models also proved that the models give consistent results of a characterized Malaysian female nasal airflow. Therefore, it was concluded that Model A represents the averaged Malaysian female nasal cavity.

## 4. Conclusions

A standardized model is required for studies involving human nasal cavities to avoid interindividual differences during comparison of results. The methodology mentioned in this research is applicable for a large group of subjects. Therefore, this is a good novelty approach to create a standardized model to represent certain populations. In addition, it is found from this research that there are clear differences between two standardized models from different geographical locations. Future work should be carried out for a larger number of test subjects to obtain a more accurate model. As a conclusion, the model generated from this study was proven to be a good and accurate representation of the adult Malaysian female nasal cavity. This new standardized model is available via corresponding author for various fields of researches. 

## Figures and Tables

**Table 1:** Table of information for 26 female subjects in current study.

Subject	Age	Race	Length, mm	Distance between slices, mm	Used for comparison
1	37	Indian	98.44	2.5	Yes
2	35	Indian	98.58	2.5	No
3	34	Chinese	104.92	2.5	No
4	38	Indian	103.48	2.5	No
5	39	Chinese	96.82	2.5	No
6	24	Chinese	107.93	2.5	No
7	43	Chinese	99.63	2.5	No
8	37	Chinese	97.61	2.5	No
9	40	Chinese	103.31	2.5	No
10	34	Indian	99.55	2.5	No
11	23	Chinese	101.80	2.5	No
12	38	Chinese	102.31	2.5	No
13	31	Chinese	98.52	2.5	No
14	20	Malay	96.05	2.5	No
15	24	Malay	94.28	2.5	Yes
16	36	Malay	94.66	2.5	No
17	40	Malay	90.86	2.5	No
18	39	Indian	99.08	2.5	No
19	32	Indian	97.92	2.5	No
20	43	Indian	100.69	2.5	Yes
21	21	Chinese	87.08	2.5	No
22	34	Malay	95.34	2.5	No
23	34	Malay	105.81	2.5	No
24	31	Chinese	94.72	2.5	Yes
25	45	Malay	87.72	2.5	No
26	40	Malay	102.06	2.5	No
Min	20	—	87.08	—	—
Max	45	—	107.93	—	—
Median	36	—	98.55	—	—
Average	34	—	98.43	—	—

**Table 2:** Contours of velocities for both models.